# Cancerous and Non-Cancerous Brain MRI Classification Method Based on Convolutional Neural Network and Log-Polar Transformation

**DOI:** 10.3390/healthcare10091801

**Published:** 2022-09-19

**Authors:** Ferdaus Anam Jibon, Mayeen Uddin Khandaker, Mahadi Hasan Miraz, Himon Thakur, Fazle Rabby, Nissren Tamam, Abdelmoneim Sulieman, Yahaya Saadu Itas, Hamid Osman

**Affiliations:** 1 Department of Computer Science and Engineering, University of Information Technology & Sciences (UITS), Dhaka 1000, Bangladesh; 2 Centre for Applied Physics and Radiation Technologies, School of Engineering and Technology, Sunway University, Bandar Sunway 47500, Selangor, Malaysia; 3 Department of General Educational Development, Faculty of Science and Information Technology, Daffodil International University, DIU Rd, Dhaka 1341, Bangladesh; 4 Department of Business Analytics, Sunway University, Bandar Sunway 47500, Selangor, Malaysia; 5 Department of Electrical Electronic & Communication Engineering, Military Institute of Science & Technology (MIST), Dhaka 1000, Bangladesh; 6 Department of Computer Science and Engineering, Sheikh Fazilatunnesa Mujib University (SFMU), Jamalpur 2000, Bangladesh; 7 Department of Physics, College of Sciences, Princess Nourah Bint Abdulrahman University, Riyadh 11671, Saudi Arabia; 8 Department of Radiology and Medical Imaging, College of Applied Medical Sciences, Prince Sattam Bin Abdulaziz University, Al-Kharj 11942, Saudi Arabia; 9 Department of Physics, Bauchi State University Gadau, PMB 65, Gadau 751105, Nigeria; 10 Department of Radiological Sciences, College of Applied Medical Sciences, Taif University, Taif 21944, Saudi Arabia

**Keywords:** convolutional neural network, log-polar transformation, principal component analysis, classification, segmentation, MRI

## Abstract

Magnetic resonance imaging (MRI) offers visual representations of the interior of a body for clinical analysis and medical intervention. The MRI process is subjected to a variety of image processing and machine learning approaches to identify, diagnose, and classify brain diseases as well as detect abnormalities. In this paper, we propose an improved classification method for distinguishing cancerous and noncancerous tumors from brain MRI images by using Log Polar Transformation (LPT) and convolutional neural networks (CNN). The LPT has been applied for feature extraction of rotation and scaling of distorted images, while the integration of CNN introduces a machine learning approach for the tumor classification of distorted images. The dataset was formed with images of seven different brain diseases, and the training set was formed by applying CNN with the extracted features. The proposed method is then evaluated in comparison to state-of-the-art algorithms, showing a definite improvement of the former. The obtained results show that the machine learning approach offers better classification with a success rate of about 96% in both plain brain MR images and rotation- and scale-invariant brain MR images. This work also successfully classified T-1 and T-2 weighted images of neoplastic and degenerative brain diseases. The obtained accuracy is perfected by several kernel procedures, while the combined performance of the two wavelet transformations and a strong dataset make our method robust and efficient. Since no earlier study on machine learning approaches with rotated and scaled brain MRI has come to our attention, it is expected that our proposed method introduces a new paradigm in this research field.

## 1. Introduction

Brain tumor detection from MRI images works based on either coefficients of the transformed domain [1] or spatial values of an image [2]. Classification of brain MRI images plays a vital role in the analysis and interpretation of brain diseases. Many methods have been proposed to design an accurate classifier to distinguish between normal and abnormal brain MRIs [3,4,5,6]. Feature extraction is a prominent process extensively used to classify brain MRIs. The extraction of features means reducing the dimensionality of the input image and transforming the simplified set of data for calculation. The process of feature extraction eliminates redundant data by measuring certain image properties. The extracted features provide relevant properties of the image into feature vectors and distinguish one pattern from another pattern [7,8,9,10].

The brain MRI is subjected to the image segmentation technique to cluster the image into simple and meaningful regions with common features and attributes. The features used for segmentation largely depend on the process of feature extraction. The image intensities are the most common feature of tumor segmentation of brain MRIs. The image pixels are grouped according to intensity level. Segmentation can therefore identify the infected region of the brain [8,9,10].

Convolutional Neural Network (CNN) is a framework in Machine Learning which takes in visual data (image) as input, and assigns weight to different features of the image while also differentiating between them. When compared to other classification methods, the amount of pre-processing required by a CNN is substantially smaller. While basic approaches require hand-engineering of filters, CNNs can learn these filters with enough training.

CNN is characterized by a finite set of processing layers that can learn various features of input data (e.g., image) with multiple levels of abstraction. Initial layers learn and extract high-level features with lower abstraction, and the deeper layers learn and extract low-level features with higher abstraction. CNN is composed of multiple building blocks known as layers of the architecture, e.g., convolutional layer, pooling layer, fully connected layer, etc. At the stage of convolutional layers, it applies a set of convolutional kernels which become convolved with the input image of N-dimensional metrics to generate an output feature map. Pooling layers are used to sub-sample the feature maps produced after convolution operations, where the larger-size feature maps are taken and shrunk to lower-sized feature maps. The last layer of CNN is called the Fully-Connected layer, where each neuron inside a layer is connected with each neuron from its previous layer; this is used as the output layer (classifier) of the CNN architecture. The activation function decides whether a neuron will fire or not for a given input by producing the corresponding output.

As far as we are concerned, no earlier studies on the machine learning approach with rotated and scaled brain MRI images are available in the literature. Therefore, in order to identify brain abnormalities from rotated and scaled brain MRI images, this paper aims to develop an improved classification technique for brain MRI images, focusing on feature extraction. The proposed method firstly applies to the feature extraction technique. Next, it presents a feature extraction method relying on Log-Polar Transformation (LPT) paired with a classification algorithm utilizing CNN to detect the brain tumor. In this way, the proposed method is also able to extract features from both T-1 and T-2 distorted brain MRI images by using LPT, which integrates CNN to initiate a machine learning approach for cancerous and noncancerous tumor classification. The scheme, thereafter, classifies the abnormal brain images and records them as either benign or malignant tumors.

The rest of the paper is organized as follows. All related works are described in Section 2. The proposed algorithm is presented in Section 3. Finally, the experimental evaluation and conclusion are presented in Section 4 and Section 5.

## 2. Related Works

Over the last decade, many methods have been proposed for tumor detection using brain MRI image segmentation. In 2012, Zhang and Wu et al [1] performed a study entitled “An MR brain images classification via principal component analysis and kernel support vector machine”. In this study, Discrete Wavelet Transformation (DWT), Principal Component Analysis (PCA), and Kernel Support Vector Machine (KSVM) were used as technical features to classify brain tumors. However, there was no direction regarding the classification of rotated and scaled brain images. In 2019, Jibon et al. [2] proposed a method for tumor detection and classification from rotated and scaled brain MRI images using log polar transformation, but no machine learning approach was applied. In continuation of that work, we have proposed a method for tumor classification from distorted brain MRI images using machine learning concepts by integrating CNN with log polar transformation. Sarhan [3] introduced wavelet transformation and CNN based-brain tumor detection and classification, but this method has produced no indication about the classification of the distorted image. Fayaz et al. [5] proposed DWT and CNN-based brain MRI classification methods, but this method was not able to identify the problem of rotated and scaled brain MRI images. Suganya et al. [6] described a method of geometric distortion of brain MRI for tumor detection and segmentation, but there was no guideline for rotated and scaled brain MRI images. Gurusamy et al. [7] introduced a method for brain tumor classification by using a machine learning approach, but this work has no indications for distorted brain tumor classification. The authors applied wavelet transformation for feature extraction, and binary tree support vector machine was implemented for classification procedure, but this work was not applicable for any types of rotated and scaled brain MRI tumor classification. Zhang et al. [11] were able to reduce the dimension of extracted features by employing wavelet transformation. They applied the K-fold stratified cross-validation method for improving the KSVM generalization. Das et al. [12] proposed a brain tumor classification method using CNN by focusing on classifying brain tumors in T1-weighted contrast-enhanced MRI images. However, this work lacked proper guidelines for using CNN in distorted brain MRI images, whereas our proposed method integrates CNN with LPT and classifies both T-1 and T-2 weighted plain and distorted brain MRI images. In [13], the authors presented a k-means cluster-based brain tumor MRI image segmentation and detection approach, where the K-means clustering algorithm and morphological filtering were used for segmentation and tumor detection, respectively, from the brain MRI images. The authors of [14] presented parallel K-means clustering for brain cancer detection with hyperspectral pictures. K-means segmentation was used as an unsupervised learning algorithm by Dhanalakshmi et al. [15]. In this method, the distance between each pixel and the K-cluster center is determined using a simple Euclidean function, and the algorithm focuses on minimizing the variance between each pixel to the cluster center in an iterative fashion. The use of principal component analysis and K-means clustering together with super pixels to distinguish tumor and non-tumor from PET scan pictures was introduced in [16]. DWT and Fuzzy C Means-based segmentation of brain MRI images were offered by Minajagi et al. [17], where PCA was further processed using SVM for the classification of only T-2 weighted brain MRI images. Deshmukh et al. [18] presented feature extraction from horizontal (LH) and vertical (HL) sub-bands of the 2D-DWT using GLCM and SVM-polynomial classifier to categorize the image as normal or abnormal. An automatic classification system of brain images in MRI based on a dual-tree complex wavelet transform and twin support vector machine was offered by [19]. In [20], a hybrid approach for the classification method of brain MRI images for tumor detection was introduced. This hybrid technique employs DWT and a Genetic algorithm for feature extraction and minimization of the number of features, followed by SVM for brain tumor classification. However, this method extracts the value of just five feature parameters, which is insufficient for sensitive categorization such as a brain tumor. A CNN-based approach to classify MRIs for brain tumors was discussed in [21]. Only the three most prevalent forms of brain tumors were classified in this method (namely Glioma, Meningioma, and Pituitary), but it cannot separate cancerous and non-cancerous states from a brain MRI image. The study [22] suggested a data augmentation-based brain tumor classification approach, whilst [23] used fuzzy C–Mean Clustering and CNN to identify brain MRI images. These models work successfully on normal MRI images for the identification of brain tumors. However, they are generally unsuccessful if the images are rotated and scaled. An improved version of the previous methods that can overcome this limitation was discussed in [24] which utilized LPT to reduce the effects of rotation and scale changes. This method discards row shift effects by introducing the resulting images to adaptive row shift-invariant wavelet transform and produces row-shifted log-polar images. However, this method performs poorly with infected images, despite acceptable results with normal ones.

From this review and analyses, we can see that there has been a lot of work on tumor classification from brain MRI images. However, no proper machine learning approach for brain tumor classification from distorted images has come to our attention. The present method that we introduce here is effective against both T-1 and T-2 weighted MRI images, alongside rotation- and scale-invariant MRI images. The accuracy of feature extraction from brain MRI images is affirmed by wavelet-based transformation (LPT), and appropriate classification is endorsed by the CNN model.

## 3. Proposed Method

This work is a follow-up study of recent work by Jibon et al. on classifying rotated and scaled brain MRI tumor images. To accomplish this task, Log Polar Transformation (LPT) was applied with Discrete Wavelet Transformation (DWT), and we used Independent Component Analysis (ICA) instead of Principal Component Analysis (PCA). However, in this work, we used the machine learning (CNN) approach to study a wide range of image categories (such as rotation- and scale-invariant MRI images as well as T-1 and T-2 weighted MRI images). 

No existing work on machine learning approaches with rotated and scaled brain MRI has come to our attention. We extracted 13 features of the brain MRI (both normal and abnormal) using LPT for rotated and scaled (distorted) brain MRI images and prepared a training set using CNN with these features, which ensured the machine learning approach. Our proposed method combining the LPT and CNN can distinguish cancerous and non-cancerous tumors from rotated and scaled brain MRI images convincingly, as perceived by a machine learning paradigm.

**Figure 1:**
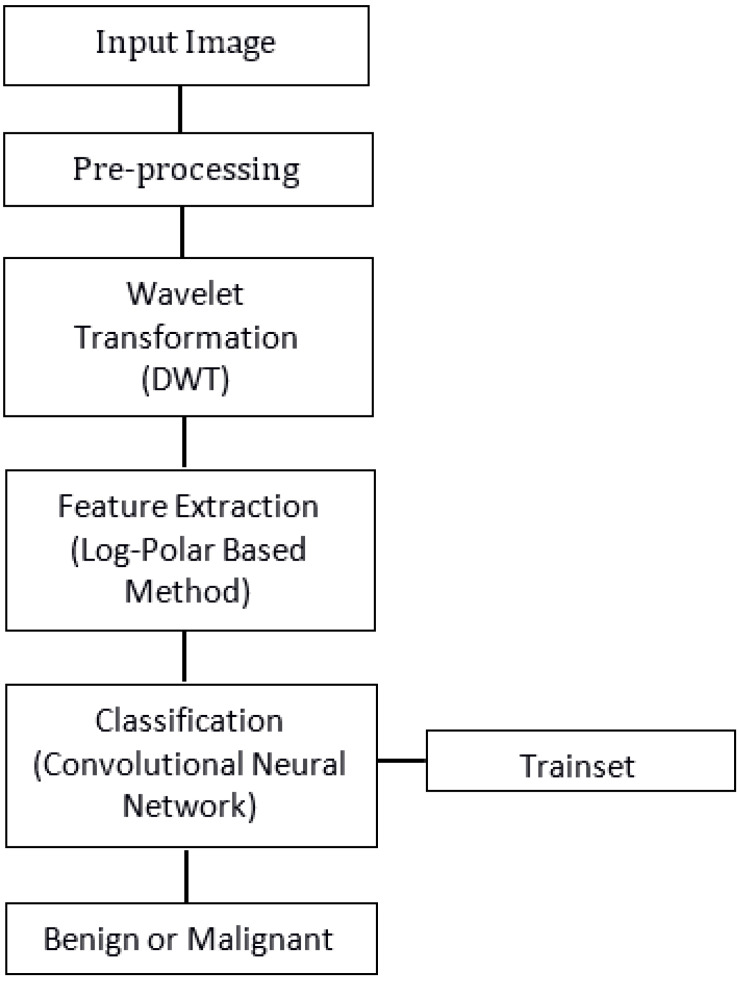
Flowchart of our proposed Algorithm.

Detection of an MRI brain tumor plays an important role in saving lives. Doctors can miss the abnormality due to inexperience in the field of tumor detection. Detection of a tumor by the proposed method is mainly divided into pre-processing, feature extraction, segmentation, and classification. Due to the poor quality of the acquired picture, pre-processing is the most important stage in the evaluation of MRI brain images. In this phase, the image is resized and converted from RGB to grayscale by eliminating the hue and saturation, while enhancing the luminance. The noise is removed to enhance the quality of the finer details of the image. A K-means clustering-based segmentation method is used in the scheme to segment the image. The proposed LPT-based feature extraction method then extracts features from the brain MRI image. The classification technique detects the tumors at the final phase of the process. Figure 1 presents the flowchart and details of the proposed algorithm.

The dataset used in this paper is The Whole Brain Atlas dataset from Harvard Medical School (https://www.med.harvard.edu/AANLIB/, accessed on 27 March 2022). This dataset contains both normal and abnormal brain images, where the category of abnormal images is formed by combining many types of brain diseases, e.g., Glioma, Meningioma, Sarcoma, etc. The multidimensional and extensive research scope of brain disease analysis motivated us to work on this dataset. In this paper, we extract features from the images of the dataset and classify brain abnormalities which are essential in achieving our goal of distinguishing between cancerous and non-cancerous brain diseases.

### 3.1. Pre-Processing

The brain MRI picture is resized for the input image at the start of the process. This input image is necessarily converted from RGB into grayscale. The process removes the hue and saturation properties. The luminance is unchanged. The grayscale image is then converted to a binary image, which assigns a value of 1 (white) to all pixels with a greater brightness level and a value of 0 to all other pixels (black).

### 3.2. Segmentation

Image segmentation is carried out to identify regions of interest and items from the image. Segmentation partitions the image into divisions or clusters to identify the target features, and it can be done via various segmentation methods, such as (i) histogram-based methods, (ii) cluster methods, (iii) edge detection methods, (iv) region growing methods, etc. Image segmentation divides the image into mutually exclusive and exhaustive pieces. It means that each segment of interest is spatially contiguous, and pixels within the segment are homogeneous concerning a predefined criterion. For segmentation, a well-known K-Means clustering technique was applied. With the help of the segmentation process, the details of the brain image have been explored in our experiment.

### 3.3. Feature Extraction

Image processing carries out feature extraction and reduction, which are essential in order to reduce complexity, data, memory, and time. In general, there are three categories of characteristics in brain MRI images: (i) characteristics based on shape (area, perimeter, circularity, irregularity shape index, etc.), (ii) characteristics based on intensity (mean, variance, standard deviation, median, skewness, kurtosis, range, pixel orientation, etc.), (iii) features based on texture (contrast, correlation, entropy, energy or uniformity, cluster shade, inverse different movement, inertia, cluster prominence, etc.) [25]. The proposed LPT-based feature extraction method works as a scale- and rotation-invariant feature extractor. The multi-resolution analytic property of DWT is utilized. To reduce the dimension of feature vectors and the computational cost of new data, principal component analysis (PCA) was applied as an excellent tool for feature reduction. Through the feature extraction process, we identified the image characteristics of cancerous and non-cancerous tumors by thoroughly analyzing the extracted features.

#### 3.3.1. Discrete Wavelet Transform (DWT)

Discrete Wavelet Transform transforms a wavelet guided by a discrete set of wavelet scales and translates it following some specific precedent. The wavelet transform formulates the signal into a mutually orthogonal set of wavelets, which is the prime difference from the continuous wavelet transform (CWT).

The proposed method uses the DWT coefficient to derive the wavelet coefficient from brain MR images. DWT produces localized frequency data that is scaled and modified from accurate wavelets. The fundamental wavelet formation is defined as follows: 

If x(t) is a square integrating function, the continuous wavelet transformation of x(t) corresponding to a specific wavelet (t) is created as follows:

(1) $$W_{\Psi }\left(a,b\right)=\overset{}{\underset{}{\int }}x\left(t\right)\Psi_{a,b}\left(t\right)dt$$

Then,

(2) $$\Psi_{a,b}\left(t\right)=\frac{1}{\sqrt[{}]{a}}\Psi \left(\frac{t-a}{b}\right)$$

‘a’ and ‘b’ are the translation and dilation parameters, respectively, in Equation (2) (both real positive numbers). Through translation and dilation, the wavelet $\Psi_{a,b}\left(t\right)$ is determined from the original wavelet Ψ(t). Equation (2) can be discretized by constraining ‘a*’* and ‘b*’* to a discrete lattice (a = 2^b^ and a > 0) to deliver the DWT, which can be presented as follows:

(3) $$ca_{j,k}\left(n\right)=DS\left[\overset{}{\underset{}{\sum }}x\left(n\right)g_{j}\left(n-2^{j}k\right)\right]$$

(4) $$cd_{jk}\left(n\right)=DS\left[\overset{}{\underset{}{\sum }}x\left(n\right)h_{j}\left(n-2^{j}k\right)\right]$$

The proposed algorithm uses DWT for composing a single-level 2D image, resulting in four sub-band (LL, LH, HL, HH) images at each scale. LL sub-band is reused for the next 2D DWT as it is considered as an approximation element of the image, while the LH, HL, and HH sub-bands are noted as the detailed elements of the image. Then, using a simple hierarchical image framework, the wavelet provides an image interpretation. The application of Discrete Wavelet Transform (DWT) tools in image processing make it easier to distinguish patterns in experimented brain images.

#### 3.3.2. Log-Polar Transformation (LPT)

Log Polar Transformation (LPT) was used in our experiments to extract features from rotated and scaled brain images appropriately. The LPT was utilized to convert the image geometry to the log-polar domain instead of the original Cartesian domain. It is an effective and ideal method because it can retain the rotation- and scale-invariant properties. The polar coordinates (r,θ) can be used to express the radius distance and angle from the center, respectively.

(5) $$\left(r,\theta \right)=\sqrt[{}]{{\left(x-x_{c}\right)}^{2}-{\left(y-y_{c}\right)}^{2}},\;tan^{-1}\frac{\left(y-y_{c}\right)}{\left(x-x_{c}\right)}$$

The following are the formulas for translating log-polar coordinates to Cartesian coordinates:

*x* = *e^p^*
*cosθ* (6)

*y* = *e^p^*
*sinθ* (7)

The LPT is a conformal mapping from Cartesian coordinates (x, *y*) to log-polar coordinates (log(r), θ).

*(log((αx), log((αy) = ((logα + logx),(logα + logy))* (8)

However, after translating the original image by (Δx, Δ*y*), the corresponding log-polar coordinates are represented by

(9) $$r^{\prime }=log\sqrt[{}]{{\left(e^{p}cos\theta -∆x\right)}^{2}+{\left(e^{p}sin\theta -∆y\right)}^{2}}$$

(10) $$\theta =tan^{-1}\frac{\left(e^{p}sin\theta -\Delta y\right)}{\left(e^{p}cos\theta -\Delta x\right)}$$

This log-polar domain is useful for extracting image features. 

#### 3.3.3. Independent Component Analysis (ICA)

ICA is primarily used as a tool for feature extraction, where we can decide which features are needed for the experiment and which one can be ignored. In our proposed method, we used ICA basically as an extension of Principal Component Analysis (PCA). PCA is a common statistical technique which orthogonally transforms the data into a new coordinate system. It can also be used to identify the variances that help remove the elements that least affect the dataset fluctuation. While PCA can only negotiate the second-order derivative of features, ICA can also deal with both second- and higher-order derivative features, making the feature engineering process more appropriate, and less important features can be easily identified and discarded. As a result, feature extraction applying ICA is more meaningful and efficient than feature extraction using PCA. The process of ICA is statistically independent and linear, but not specifically orthogonal. 

### 3.4. Classification

Classification can be described as a technique to distinguish, differentiate, or separate the patterns of an arbitrary image by partitioning meaningful and distinct clusters, and pinpointing a homogeneous pixel of common image class characteristics. There are two conventional ways for classification: (i) unsupervised classification (K-Means, Fuzzy C-Means, Self-Organization Feature Map, Ant Tree Algorithm, Expectation Maximization, Hierarchical Clustering, etc.) and (ii) supervised classification (K-Nearest Neighborhood, Support Vector Machine, Principle Component Analysis, Bayes Classifier, etc.) [25]. For the spectral characteristics of pixels or classes inside the multispectral feature space to be identified as identical units, unsupervised classification is performed. However, supervised classification uses known identity samples to identify unknown identities. To choose the nature of the supervised classification of the proposed method, classification accuracy was considered. After evaluating the success classification rate, Kernel Support Vector Machine (KSVM) was decided. [26]. A convolution neural network (CNN) is a type of image classification technique that is a hybrid of machine learning and neural networks. CNN learns features from input data using 2D convolutional layers. This suggests that this network is well suited to two-dimensional image processing. In comparison to other image classification methods, CNN requires an extremely minimal setup. This implies that they can learn the filters that would otherwise have to be constructed by hand in other algorithms. Image and video recognition, image classification, and recommender systems, as well as natural language processing and medical image analysis, can all benefit from CNNs.

#### Convolutional Neural Network (CNN)

The architecture of a CNN is inspired by the organization of the visual cortex, which is equivalent to the connectivity pattern of neurons in the human brain. Convolutional neural networks’ distinctive characteristic is their ability to learn a vast number of filters in parallel, unique to a training dataset, within the constraints of a specific predictive modeling problem, such as image classification. CNN has four layers, including an input layer, a convolution layer, a pooling layer, and a fully connected layer.

Input such as [32 × 32 × 3] contains the raw pixel values of the image. In this case, it is an image with 32 widths, 32 heights, and three colors channels (R, G, and B). The convolution layer will compute the output of neurons connected to particular regions in the inputs, with each computing a dot product between respective weights and a little region connected to it in the input volume. If we chose to employ 12 filters, it might result in a volume of [32 × 32 × 12]. The RELU layer applies a per-element activation function from zero to a threshold max (0, x). This will not change the volume size ([32 × 32 × 12]). The pooling layer will reduce the volume to [16 × 16 × 12] by downsampling along the spatial dimensions, i.e., the width and height. The class scores will be computed by the fully-connected layer, resulting in a volume of size [1 × 1 × 10], where each of the ten numbers corresponds to a class score, such as among the CIFAR-10 categories. As with regular Neural Networks, as the name implies, each neuron in this layer will be connected to all of the neurons in the preceding volume. It is necessary to provide the filter’s sliding stride. The filters are shifted one pixel at a time when the stride is set to 1. When the stride is set to 2, the filters will jump two pixels at a time as we move them around. As a result, the spatial output volumes will be reduced. On occasion, padding the input volume with zeros around the border can be advantageous. This zero-size padding is a hyperparameter. Zero padding has the advantage of allowing us to control the spatial size of the output volumes.

**Figure 2:**
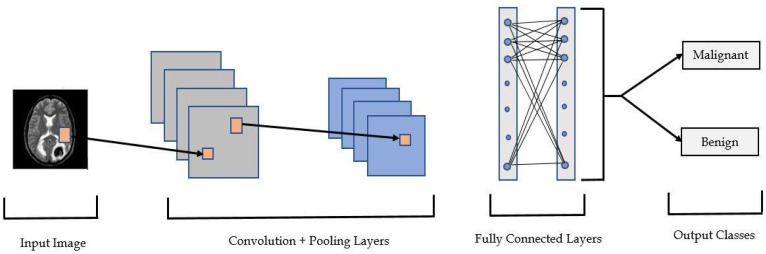
Convolution Neural Network for Tumor Image Classification.

Finally, we used a fully connected dense layer with a certain number of neurons, as well as the softmax output layer, to calculate the probability score for each class and classify the final decision labels, indicating whether the input MRI image contains cancer or not. Figure 2 depicts the relevant modified version of CNN used in our work.

## 4. Experiment

Finally, we used an appropriate experimental setup to apply our categorization algorithm. There are three steps to our experiment. We start by organizing a well-organized dataset, then designing the simulation method, and then simulating and observing the pattern of the results. The following is a brief description of our experimentation approach.

### 4.1. Dataset Formation

We formed a well-organized dataset utilizing the resource provided by the Harvard Medical School (http://www.med.harvard.edu/AANLIB/, accessed on 13 September 2022). The dataset includes both T-1 weighted and T-2 weighted MRI images. T-1 weighted images produce fat bright tissue types. Repetition Time (TR) and Time to Echo (TE) are short for T-1 weighted images during MRI processing. In contrast, T-2 weighted images produce two bright tissue types: fat and water. TR and TE are long.

Both T-1 and T-2 weighted images are rotated and scaled if needed (in the case of rotation- and scale-invariant images). We changed the orientation with a rotation factor of −1800 to +1800, and shifted the scale using a scaling factor of 1.5.

The collection of contaminated brain MRI images includes cases of Glioma, Meningioma, Sarcoma, Alzheimer’s disease, Huntington’s disease, Pick’s disease, and Alzheimer’s disease combined with visual agnosia.

The classification benchmark shows that the brain tumor is either noncancerous (Benign) or cancerous (Malignant). Here, we take 48 T-2 images, 24 T-1 images, and 20 rotation- and scale-invariant images (Table 1, Table 2 and Table 3). We determine the two categories of brain tumors from our training dataset of selected images, which extracted the values of significant thirteen (13) parameters of brain MRI images.

### 4.2. Design Simulation

MATLAB 2014b has been used to build the simulation process in order to formulate our methodology. The input picture formats for our experiment are *.jpg, *.png, and *.bmp. The input image must then be transformed to a color space that is device independent. Colors in the a*b* color space can be classified using the K-means clustering algorithm. The image now has three colors and creates three clusters. The pixel distances in the cluster are calculated using the Euclidean distance metric. We labeled every pixel in the image using the K means clustering results. We needed to store the results of the clustering. Thus, a blank cell array was created. An RGB label was also made using pixel labels.

In the case of LPT, it also transforms a conventional image into a polar form. This procedure produces an image of the dimensions M × N. Here, M points are along the r axis, and N points are along the theta axis (assuming the origin is at the center). We interpolated between any points that are not located within the image using bilinear interpolation.

Our next goal was to utilize DWT and LPT to extract features from a tested image. We applied PCA and thirteen features were extracted from the given image. These features include (1) contrast, (2) correlation, (3) energy, (4) homogeneity, (5) mean, (6) standard deviation, (7) entropy, (8) RMS, (9) variance, (10) smoothness, (11) kurtosis, (12) skewness, and (13) inverse difference movement (IDM). By processing every image, thirteen values were obtained. Based on these values, the training dataset was created. The training dataset for tumor classification of T-2 weighted images was comprised of a 38 × 13 matrix. For the training dataset of T-1 weighted images, we used the 18 × 13 matrix; for simulated images, the 16 × 13 matrix was used.

This project entails developing a CNN-based shallow model for brain MRI image classification from the ground up. The straightforward four convolutional layers CNN (4L-CNN) is made up of four convolutional layers, two fully connected layers, a dropout layer, and an output layer for determining whether a tumor is benign or malignant. After four successive convolutional layers, a dense layer network is built using four fully linked layers. The flattened output of the convolutional network is received by the first fully connected layer, which is made up of 256 ReLU active nodes. The second fully connected layer is also ReLU activated and contains 512 neurons that receive a 256-dimensional vector from the previous layer’s output. The third fully connected layer, with 512 nodes, is a dropout layer with a 50% dropout. Dropout, which randomly ignores some neurons in training, prevents overfitting. Finally, the output of the dropout layer is fed into a softmax activated output, which assigns each class a probability. Dropout assigns a probability of 0.5 to each hidden neuron producing zero output. As a result, the neurons that have been “dropped out” do not contribute to the forward or backward pass during training. Dropout is used in the layer just before the output layer. With a batch size of 10 samples and a learning rate of 0.00001, we used the Root Mean Square Propagation optimizer (RMSprop). The images were rescaled to 224 × 224 dimensions, and each input has three (RGB) channels. Finally, we tested the validation dataset based on the training dataset and classified the validation dataset correctly. Four basic image analytic kernel operations were used to derive the classification accuracy: (i) RBF kernel, (ii) LINEAR kernel, (iii) POLYNOMIAL kernel, and (iv) Quadratic Kernel.

There was no padding, and stride 1 was used for the convolution operation. Between the convolutional layers and after the final convolutional layer, the max-pooling layers were used. In these layers, the pool size and stride were both two. The pooling layers, like the convolutional layers, did not have any padding.

### 4.3. Simulation and Observation/Output of Simulation

**Figure 3:**
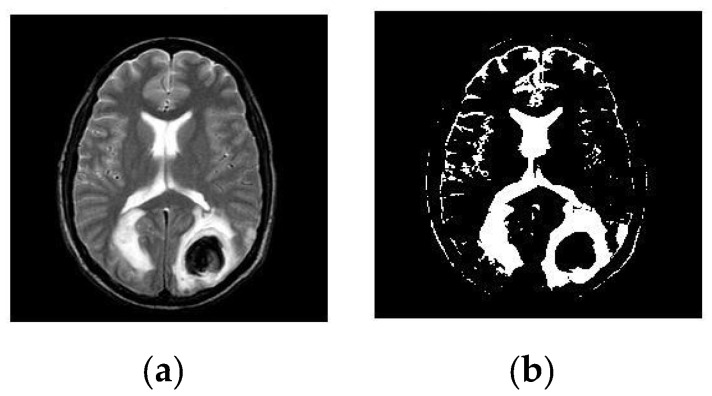
Here, image (**a**) is an input T-2 brain MRI image, whereas (**b**) is a segmented T-2 image. It is a Malignant Tumor according to the segmented picture.

**Figure 4:**
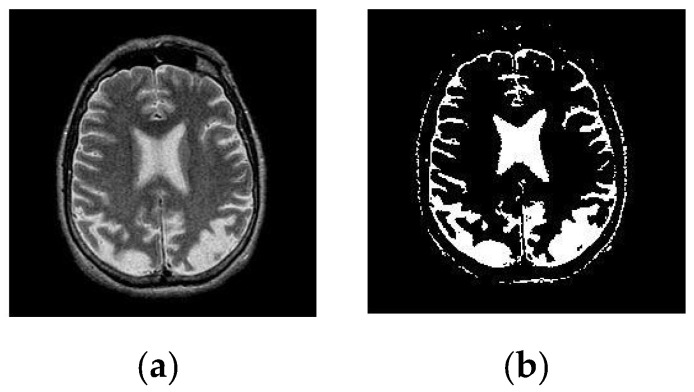
Here, image (**a**) is the input T-1 brain MRI image and image (**b**) is the segmented T-1 image. A segmented image classifies it as a Benign Tumor.

**Figure 5:**
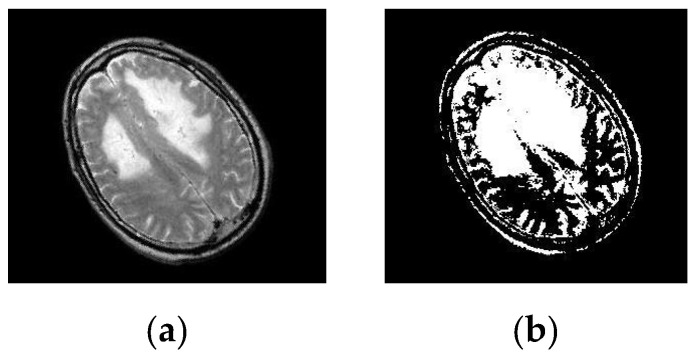
Here, image (**a**) is an input distorted/simulated (rotated and scaled) brain MRI image and image (**b**) is a segmented simulated (rotated and scaled) image. A segmented image classifies it as a Malignant Tumor.

**Figure 6:**
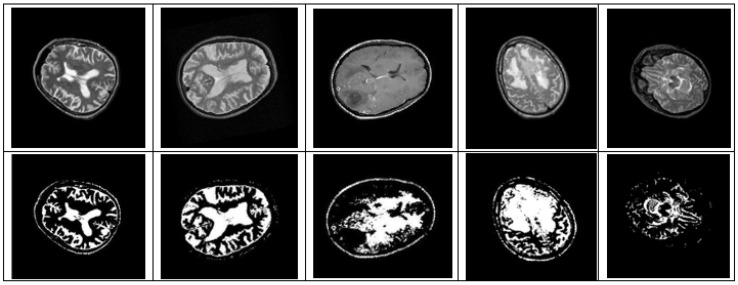
Rotation Invariant (−180° to +180°) Brain MRI Image Detected by CNN.

**Figure 7:**
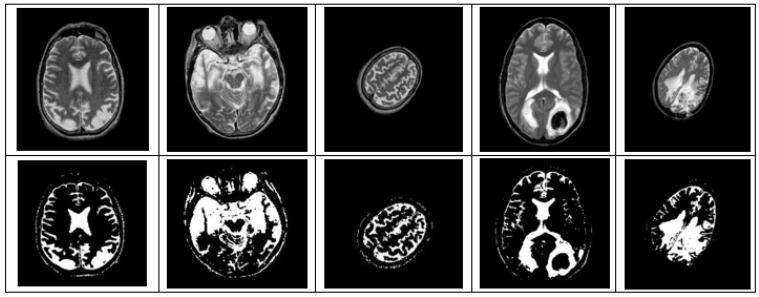
Scaling Invariant (0.25–1.5) Brain MRI Image Detected by CNN.

After simulating each image, the following result (shown in Figure 3, Figure 4, Figure 5, Figure 6 and Figure 7) was obtained. The classification technique is demonstrated here. For tumor classification, the aim was to correct classification and observe the resulting pattern of our proposed algorithm.

## 5. Result Analysis

Our proposed method shows suitability to detect brain tumors from brain MRI images and classify the brain successfully. In the present experiment, two wavelet transforms, namely (a) Log Polar Transformation (LPT) and (b) Discrete Wavelet Transform (DWT) were applied. CNN was used for classification together with LPT. Here, we compare the performance accuracy considering four fundamental properties of image analysis: (i) RBF kernel, (ii) LINEAR kernel, (iii) POLYNOMIAL kernel, and (iv) Quadratic Kernel. The comparative analysis was accomplished on three different 256 × 256 image datasets: (i) Distorted/Simulated MRI Images, (ii) T-1 weighted Images, and (iii) T-2 weighted Images.

The precise segmentation of brain MRI scans is required for both detecting and appropriately diagnosing any anomalies in the images. The segmentation process can select abnormal brain MRI images randomly from our image dataset. The image dataset includes neoplastic and degenerative illnesses such as visual agnosia, glioma, meningioma, sarcoma, Alzheimer’s disease, Huntington’s disease, Pick’s disease, and Alzheimer’s disease. The scheme then categorizes the abnormal brain images with a probability of having cancer tissues. Nevertheless, a benign brain tumor is a slow-growing non-cancerous mass of cells and is often stationary. An appropriate surgery can remove a benign tumor safely. The malignant brain tumors most surely signify a cancer case. Malignancy spreads rapidly and aggressively affects nearby tissues. In that case, the patient usually experiences radiotherapy or chemotherapy to kill the cancerous cell. Furthermore, the malignant tumor often eventually returns after the treatment. If this happens, a cure is not possible in usual cases. Doctors then try to improve the symptoms for prolonging the patient’s life. The matter of improving the symptoms depends on the accuracy of detection. This work employs T-1 and T-2 weighted MRI images of neoplastic diseases which are rotation- and scale-invariant. It successfully detects benign and malignant tumors in the MRI images.

### 5.1. Result 1: Experiment with Distorted/Simulated MRI Image Dataset

**Figure 8:**
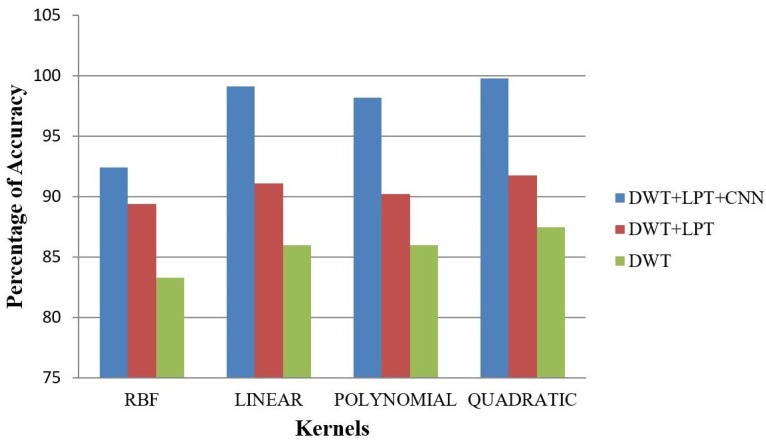
Average Accuracy for Abnormality Detection of Distorted/Simulated images.

(a) Abnormality Classification for Distorted/Simulated MRI Image:

We randomly selected 16 normal and abnormal 256 × 256 MRI images. We converted the image orientation using a rotation range of −1800 to 1800 and changed the scales using a scaling factor of 1.5. We performed our simulation over these MRI images and successfully separated normal and abnormal brains. The accuracy of the classification of brains is given in Table 4 and Figure 8.

(b) Tumor Classification for Distorted/Simulated MRI Image

**Figure 9:**
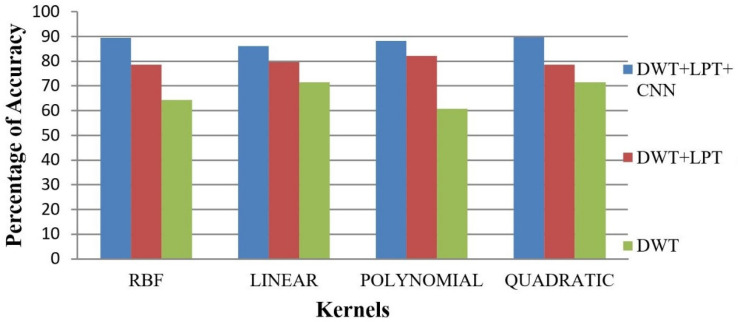
Average Accuracy for Tumor Detection of Distorted/Simulated image.

**Figure 10:**
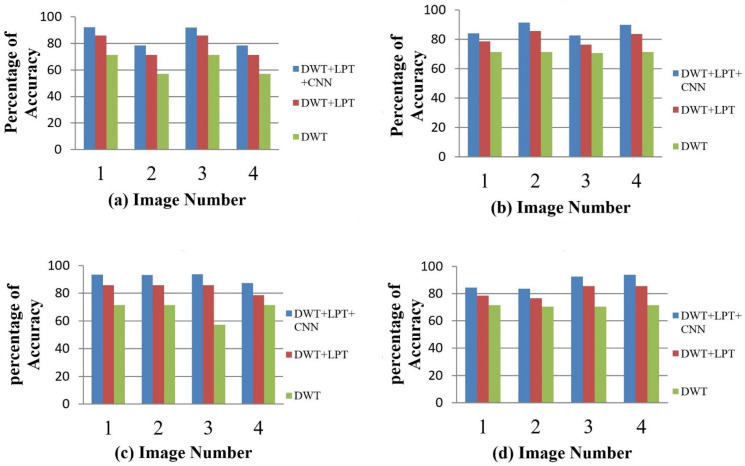
The percentage of accuracy versus image for distorted/simulated image: (**a**) RBF; (**b**) Linear (**c**) Polynomial; (**d**) Quadratic.

We selected 20 rotated and scaled images. They were used to classify brain tumors for rotation- and scale-invariant cases. Our simulation can separate benign and malignant tumors. The classification accuracy of brain tumors is given in Figure 9 and Figure 10. 

### 5.2. Result 2: Experiment with T-1 Weighted MRI Image Dataset

(a) Abnormality Classification for T-1 Weighted Images

**Figure 11:**
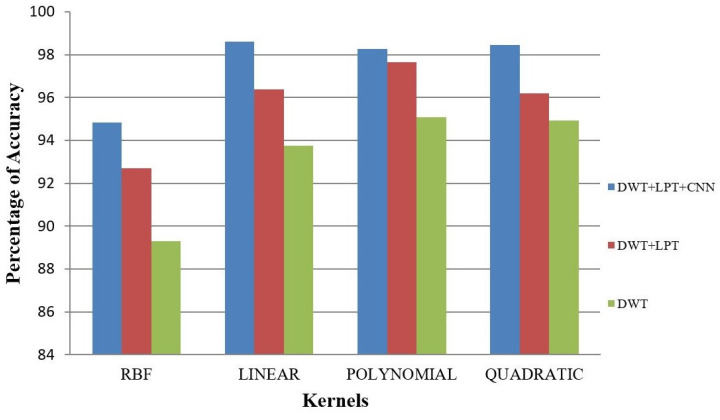
Average Accuracy for Abnormality Detection of T-1 weighted images.

The T-1 weighted image dataset consists of 24 brain MRI images that do not have high contrast. The accuracy of the classification of brains is given in Table 5 and Figure 11. They are also less clear than T-2 weighted images.

(b) **T-1 Weighted Image Tumor Classification**

**Figure 12:**
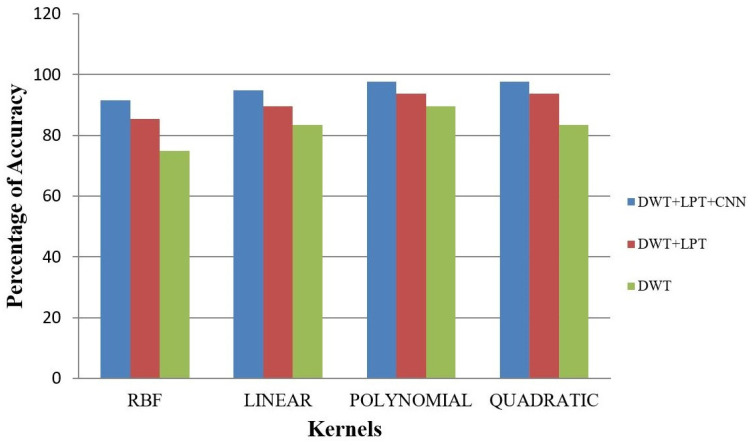
Average Accuracy for Tumor Detection of T-1 weighted images.

**Figure 13:**
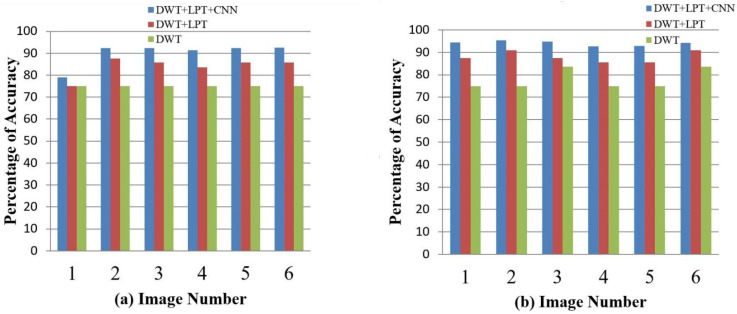

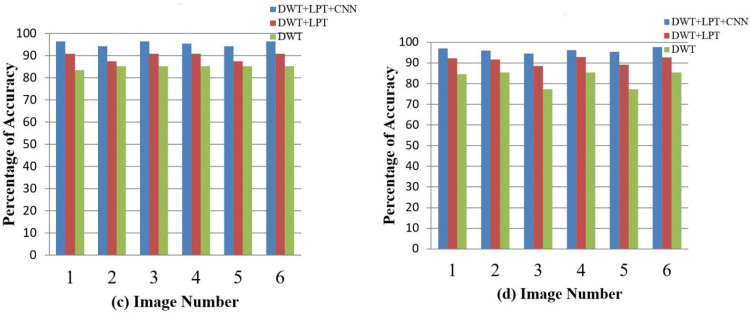
The accuracy percentages for the T-1 weighted images: (**a**) RBF; (**b**) Linear; (**c**) Polynomial; (**d**) Quadratic.

T-1 weighted images have lower image quality compared to T-2 weighted images. Hence, image classification of brain tumors for T-1 weighted images is more challenging. Here, we have selected 24 abnormal (17 benign tumors and 7 malignant tumors) T-1 brain tumor images. The result of accuracy is shown in Figure 12 and Figure 13.

### 5.3. Result 3: Experiment with T-2 Weighted MRI Image Dataset

(a) Classification of Abnormalities in T-2 Weighted Images

**Figure 14:**
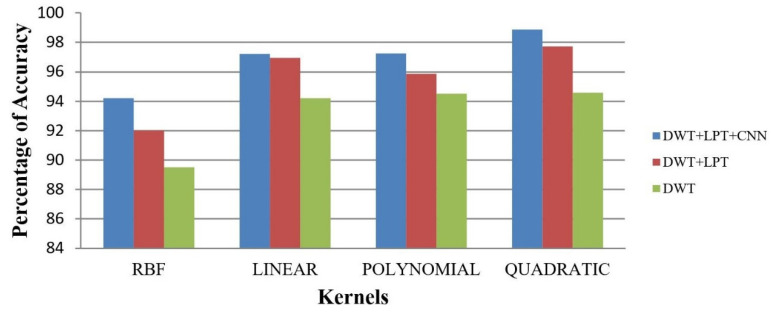
Average Accuracy for Abnormality Detection of T-2 weighted images.

**Figure 15:**
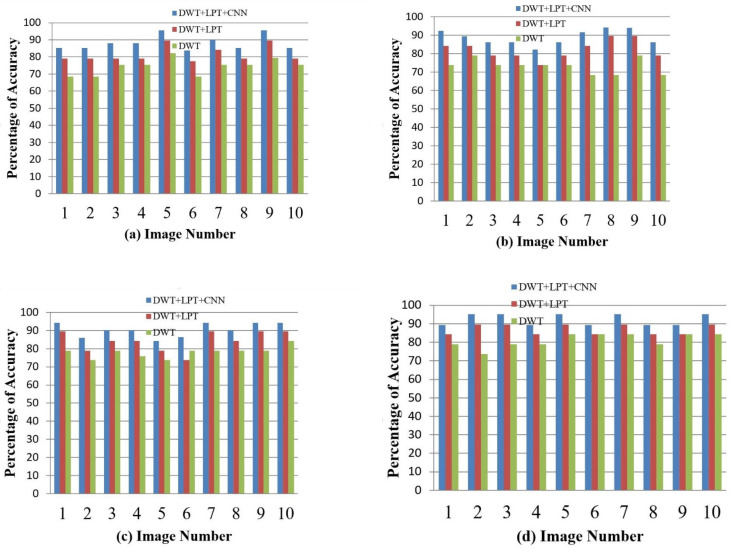
The accuracy percentages for the T-2 weighted images: (**a**) RBF; (**b**) Linear; (**c**) Polynomial; (**d**) Quadratic.

We made the T-2 weighted image dataset for abnormality classification with a total of 64 brain images. This dataset consists of 16 normal brain images and 48 abnormal brain images. We have taken seven types of disease (Glioma, Meningioma, Sarcoma, Huntington’s disease, Picks disease, Alzheimer’s disease and Alzheimer’s disease plus visual agnosia) for infected MRI images, which we called abnormal images. The result of accuracy is given in Table 6 and Figure 14 and Figure 15.

(b) Tumor Classification for T-2 Weighted Images

To classify the brain tumor as benign or malignant, we randomly selected 48 abnormal images (22 benign and 26 malignant tumor images). We selected four types of benign diseases (Huntington’s disease, Picks disease, Alzheimer’s disease, and Alzheimer’s disease plus visual agnosia) and three types of malignant diseases (glioma, meningioma, and sarcoma). The result for accuracy is as follows:

At each step of the experiment, we demonstrated that our proposed method successfully classified cancerous and non-cancerous brain tumors. Previously, we were not familiar with the application of machine learning approaches with distorted brain MRI images; hence, this work has been successful in distinguishing cancerous and non-cancerous tumors from rotated and scaled brain MRI images.

## 6. Conclusions

In this research, we have used the Convolution Neural Network for brain MRI image classification as part of a machine learning approach. We conducted several experimental evaluations to validate our proposed extraction method using a dataset comprising images from brain MRI. We have found from the experimental analysis that our developed method of classification successfully identified both cancerous and non-cancerous tumor cases from brain MRI images. It can identify tumors and classify them as benign or malignant. We used both LPT and DWT to extract correct features. ICA decreases the search space without causing the detection factor to be misinterpreted. We have successfully classified brain MRI images with rotation- and scale-invariant properties. Moreover, we also succeeded in classifying T-1 and T-2 weighted images of neoplastic and degenerative brain diseases. Our experiment’s accuracy measurement was perfected by employing four kernel procedures (RGB, LINEAR, POLYNOMIAL, and QUADRATIC). The combined performance of two wavelet transformations and a strong dataset makes our method robust and efficient. The use of LPT for rotated and scaled images and the successful application of the machine learning approach through the integration of CNN with these distorted brain images have added a new dimension to tumor classification research.

We further plan to work on cerebrovascular and inflammatory diseases. We plan to focus our analysis on various wavelet types, e.g., complex wavelet transforms (CWT), dual-tree complex wavelet transforms (DTCWT), etc. Our future focus turns into the classification of a variety of brain diseases by handling different wavelet families as well as reducing time consumption and increasing the success rate.

## 7. Limitations

The rotated images taken in our proposed method are basically able to classify the brain image rotated from −180 degrees to +180 degrees; we have not studied rotation images outside this range. We prepared our training model with the scaled images worked out from 25 percent to 150 percent, and the brain can classify the images in this scaling range only. This research has not covered brain images outside this scaling range. However, we hope to conduct a detailed study to overcome these limitations in the future.

## Figures and Tables

**Table 1:** Simulated images for the experiment.

Classification	Simulated Images			
Tumor Classification	Training		Validation	
	Benign	Malignant	Benign	Malignant
	9	7	2	2

**Table 2:** T-2 weighted images for the experiment.

Classification	T-2 Weighted Images			
Tumor Classification	Training		Validation	
	Benign	Malignant	Benign	Malignant
	18	20	4	6

**Table 3:** T-1 weighted images for the experiment.

Classification	T-1 Weighted Images			
Tumor Classification	Training		Validation	
	Benign	Malignant	Benign	Malignant
	13	5	4	2

**Table 4:** Average accuracy for Distorted/Simulated Images.

Classification	Method	Simulated Images			
		RBF (%)	Linear (%)	Polynomial (%)	Quadratic (%)
Abnormality Classification	DWT	83.29	85.97	86.37	87.49
	DWT + LPT	89.41	91.11	90.21	91.78
	DWT + LPT + CNN	92.41	99.11	98.21	99.78
Tumor Classification	DWT	64.29	71.43	60.71	71.43
	DWT + LPT	78.57	79.64	82.14	78.57
	DWT + LPT + CNN	89.41	86.11	88.21	89.78

**Table 5:** Average T-1 weighted image accuracy.

Classification	Method	T-1 Weighted Images			
		RBF (%)	Linear (%)	Polynomial (%)	Quadratic (%)
Abnormality Classification	DWT	89.29	93.75	95.09	94.94
	DWT + LPT	92.71	96.40	97.66	96.21
	DWT + LPT + CNN	94.84	98.62	98.28	98.45
Tumor Classification	DWT	75.00	83.33	89.58	83.33
	DWT + LPT	85.42	89.58	93.75	93.75
	DWT + LPT + CNN	91.48	94.83	97.66	97.66

**Table 6:** Average accuracy for T-2 weighted images.

Classification	Method	T-2 Weighted Images			
		RBF (%)	Linear (%)	Polynomial (%)	Quadratic (%)
Abnormality Classification	DWT	89.51	94.20	94.53	94.59
	DWT + LPT	92.02	96.94	95.88	97.72
	DWT + LPT + CNN	94.21	97.23	97.26	98.87
Tumor Classification	DWT	77.90	73.16	77.90	81.05
	DWT + LPT	80.53	81.05	82.63	84.74
	DWT + LPT + CNN	91.33	93.85	95.67	97.28

## Data Availability

All data are available in the manuscript.
